# Crystal structure and Hirshfeld analysis of 3′-bromo-4-methyl­chalcone and 3′-cyano-4-methyl­chalcone

**DOI:** 10.1107/S2056989020011135

**Published:** 2020-08-25

**Authors:** Zachary O. Battaglia, Jordan T. Kersten, Elise M. Nicol, Paloma Whitworth, Kraig A. Wheeler, Charlie L. Hall, Jason Potticary, Victoria Hamilton, Simon R. Hall, Gemma D. D’Ambruoso, Masaomi Matsumoto, Stephen D. Warren, Matthew E. Cremeens

**Affiliations:** a Department of Chemistry & Biochemistry, Gonzaga University, 502 E Boone Ave, Spokane, WA 99258, USA; b Department of Chemistry, Whitworth University, 300 W. Hawthorne Rd, Spokane, WA 99251, USA; c School of Chemistry, University of Bristol, Cantock’s Close, Bristol, BS8 1TS, England

**Keywords:** crystal structure, chalcone, bromo, cyano, halogen bond, π stacking, edge-to-face

## Abstract

Two crystal structures of chalcones, or 1,3-di­aryl­prop-2-en-1-ones, are presented; both contain a methyl substitution on the 3-Ring, but differ on the 1-Ring, bromo *versus* cyano. The compounds are 3′-bromo-4-methyl­chalcone [systematic name: 1-(2-bromo­phen­yl)-3-(4-methyl­phen­yl)prop-2-en-1-one], C_16_H_13_BrO, and 3′-cyano-4-methyl­chalcone {systematic name: 2-[3-(4-methyl­phen­yl)prop-2-eno­yl]benzo­nitrile}, C_17_H_13_NO. Both chalcones meaningfully add to the large dataset of chalcone structures. The crystal structure of 3′-cyano-4-methyl­chalcone exhibits close contacts with the cyano nitro­gen that do not appear in previously reported disubstituted cyano­chalcones, namely inter­actions between the cyano nitro­gen atom and a ring hydrogen atom as well as a methyl hydrogen atom. The structure of 3′-bromo-4-methyl­chalcone exhibits a type I halogen bond, similar to that found in a previously reported structure for 4-bromo-3′-methyl­chalcone.

## Abstract 2

The 3′-cyano-4-methyl­chalcone crystal structure exhibits close contacts with the cyano nitro­genatom, which do not appear in previously reported disubstituted cyano­chalcones. This structure is the first reported for a *meta*-cyano chalcone, while noting that the structure for 3′-bromo-4-methyl­chalcone is also a first.

## Chemical context   

Chalcones are organic mol­ecules commonly found in nature consisting of two phenyl rings connected by an α,β-unsaturated ketone, or enone. Inter­est in chalcone mol­ecules has risen because of their potential pharmaceutical properties, electronic properties, and straightforward synthesis *via* a Claisen–Schmidt condensation between a benzaldehyde and aceto­phenone (Zhuang *et al.*, 2017 ▸). Pharmaceutical attributes shown by some chalcones include anti­oxidant, anti-inflammatory, anti-cancer, and cytotoxic properties (Sahu *et al.*, 2012 ▸). Additionally, some chalcones have been shown to be fluorescent, making them potential probes for mechanistic investigations and imaging (Lee *et al.*, 2012 ▸).
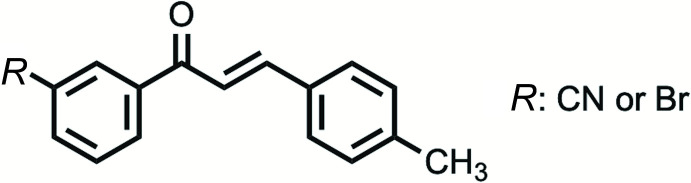


**Figure 1:**
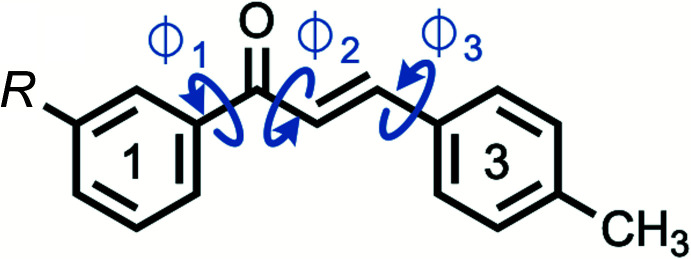
Three key dihedrals describing the chalcone planarity for 3′-cyano-4-methyl­chalcone (*m*′CN*p*CH_3_) and 3′-bromo-4-methyl­chalcone (*m*′Br*p*CH_3_); the 1-Ring and 3-Ring are labelled, where *R* = CN, Br.

This paper compares the structure and packing of two newly crystallized chalcone mol­ecules, 3′-cyano-4-methyl­chalcone [Sm6p] or *m*′CN*p*CH_3_ and 3′-bromo-4-methyl­chalcone [Dm6p] or *m*′Br*p*CH_3_, where Sm6p and Dm6p are inter­nal codes tied to a large, long-term project. Each chalcone examined consists of a variable *meta* substitution at C6 of the 1-Ring, and methyl substitution at C13 of the 3-Ring, see Figs. 1 ▸ and 2 ▸. Substitution on the 1-Ring has been utilized to further understand the packing and structure of chalcone crystals based upon their ring substituents.

## Structural commentary   

**Figure 3:**
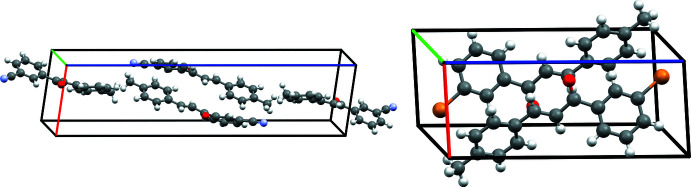
The unit cells of *m*′CN*p*CH_3_ (*P*2_1_/*c* space group, left) and *m*′Br*p*CH_3_ (*P* 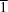 space group, right), with the *a*, *b*, and *c* axes indicated in red, green, and blue, respectively.

The chalcones under observation, *m*′CN*p*CH_3_ and *m*′Br*p*CH_3_, differ at the *meta* position on the 1-Ring, cyano and bromo respectively, Figs. 1 ▸ and 2 ▸. Note that the following summary of dihedrals, which represents the planarity of the chalcones, references data in Table 1 ▸ where non-rounded angles and errors can be found. The enone core exhibits small (10–11°) deviations from planarity (Φ2) for *m*′CN*p*CH_3_ and *m*′Br*p*CH_3_. The 1-Ring/carbonyl twists (Φ1) show similar deviations from planarity (25–27°) for *m*′CN*p*CH_3_ and *m*′Br*p*CH_3_. The 3-Ring/alkene twists (Φ3) also show similar deviations from planarity (16–18°) for *m*′CN*p*CH_3_ and *m*′Br*p*CH_3_. *m*′CN*p*CH_3_ and *m*′Br*p*CH_3_ exhibit similar 1-Ring/3-Ring twist angles (approximately 49°) and fold angles (1–2°). Based on the angle values, *m*′CN*p*CH_3_ and *m*′Br*p*CH_3_ do not vary greatly in torsions despite their different substituents. Both chalcones are similarly twisted and show a pairwise anti­parallel arrangement of the enone core (Fig. 3 ▸), which are related by inversion symmetry. A closer look at the supra­molecular properties (see below) reveals similarities and differences for the crystal structures.

## Supra­molecular features   

**Figure 4:**
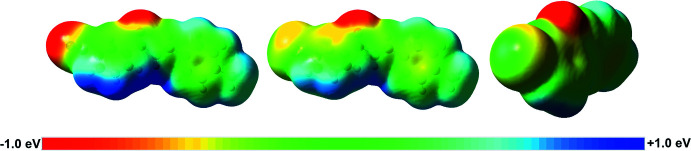
Electrostatic potentials at the wB97XD/6–311++G(d,p) level of theory for *m*′CN*p*CH_3_ (left) and *m*′Br*p*CH_3_ (middle and right). The range for all three plots is from −1.0 eV (red) to +1.0 eV (blue); electrostatic potential maps were plotted on the 0.0004 SCF density surface. Single point energy calculations were performed on the geometric coordinates of the asymmetric unit (Frisch *et al.*, 2009 ▸).

**Figure 5:**
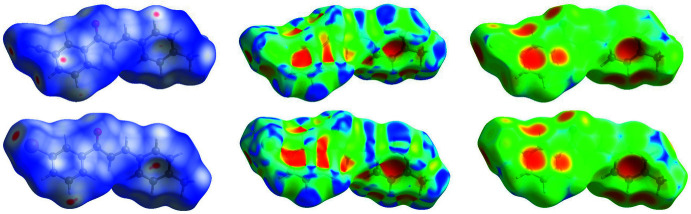
Hirshfeld surfaces of *m*′CN*p*CH_3_ (top) and *m*′Br*p*CH_3_ (bottom). Surfaces are mapped with *d*_norm_ (left), the shape-index (middle), and *d*_e_ (right). Note that close contacts involving the aromatic rings visualized in *d*_norm_ are also supported in both the shape-index and *d*_e_, as indicated by the red regions over the rings.

Electrostatic potentials are shown in Fig. 4 ▸, and Hirshfeld analyses are presented in Figs. 5 ▸–7 ▸ ▸ for *m*′CN*p*CH_3_ and *m*′Br*p*CH_3_. The electrostatic potentials show a greater polarization for *m*′CN*p*CH_3_ than for *m*′Br*p*CH_3_, which is expected because the cyano functional group is a stronger electron-withdrawing group than bromine. Consequently, the 1-Ring hydrogen atoms of *m*′CN*p*CH_3_ exhibit greater partial positive character; nonetheless, the 1-Rings for both *m*′CN*p*CH_3_ and *m*′Br*p*CH_3_ show C—H⋯π inter­actions, see discussion below. Additionally, the small and slightly positive region on Br1 (Fig. 4 ▸, right) hints toward a σ-hole and an opportunity for a halogen bond in *m*′Br*p*CH_3_. The Hirshfeld analyses below highlight the main inter­molecular inter­actions found in *m*′CN*p*CH_3_ and *m*′Br*p*CH_3_ (Spackman & Jayatilaka, 2009 ▸); see the supporting information for fingerprint plots showing the percentage distribution of the inter­molecular inter­actions represented by the *d_norm_* surface in Fig. 5 ▸.

**Figure 6:**
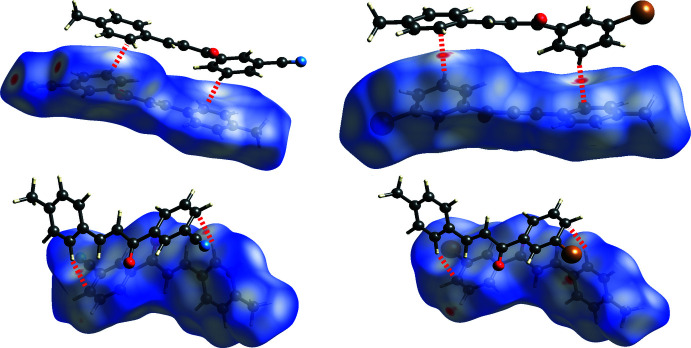
Hirshfeld short contact (*d*_norm_) plots of *m*′CN*p*CH_3_ (left) and *m*′Br*p*CH_3_ (right) showing the C8—H8⋯C11 (top) and C11—H11⋯·C7 (bottom) inter­actions. Red, white, and blue surface colors indicate contacts less than the sum of the van der Waals radii, close to, or greater than, respectively. For *m*′CN*p*CH_3_ and *m*′Br*p*CH_3_, the inter­acting chalcone mol­ecules are anti­parallel to one another with the carbonyl groups facing opposite each other.

**Figure 7:**
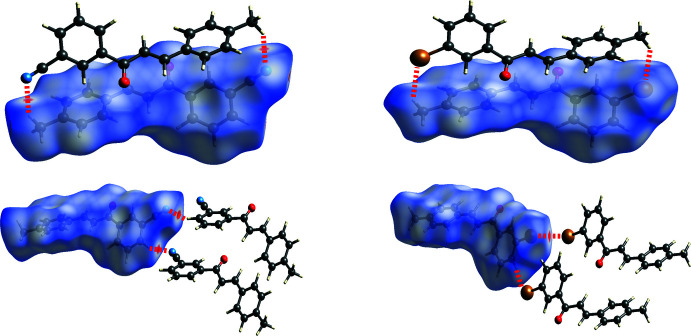
Hirshfeld short contact (*d*_norm_) plots of *m*′CN*p*CH_3_ (left) and *m*′Br*p*CH_3_ (right) showing the C17—H17*A*⋯N1 and C16—H16*A*⋯Br1 (top), as well as C7—H7⋯N1, C7—H7⋯Br1, and C6—Br1⋯Br1 (bottom) inter­actions. Red, white, and blue surface colors indicate contacts less than the sum of the van der Waals radii, equal to, or greater than, respectively. Note that the C7—H7⋯N1 inter­action for *m*′CN*p*CH_3_ involves three mol­ecules, while for *m*′Br*p*CH_3_ both C7—H7⋯Br1 and Br⋯Br1 inter­actions are needed to support a similar three-mol­ecule arrangement.

From a Hirshfeld analysis, the *d_norm_* surfaces indicate close contacts (red regions) near H7, H8, H11, H17*A*, C7, C11, and N1 for *m*′CN*p*CH_3_ and H8, C11, and Br1 for *m*′Br*p*CH_3_. Upon closer inspection of these atoms, *m*′CN*p*CH_3_ and *m*′Br*p*CH_3_ contain multiple C—H⋯π inter­actions, which can be seen in Fig. 6 ▸ as red regions. Note that the following summary of short contacts between two atoms, which have distances less than the sum of their van der Waals (vdW) radii, references data found in Table 2 ▸ where non-rounded distances and errors can also be found. Notable hydrogen–carbon short contacts for *m*′CN*p*CH_3_ are C8—H8⋯C11^iv^ (2.88 Å) and C11—H11⋯C7^iii^ (2.82 Å). In comparison, similar short contacts for *m*′Br*p*CH_3_ are C8—H8⋯C11^iii^ (2.80 Å) and C11—H11⋯C7^ii^ (2.89 Å). *m*′CN*p*CH_3_ contains some notable C—H⋯N inter­actions, which can be seen in Fig. 7 ▸ as red regions. The hydrogen–nitro­gen short contacts for *m*′CN*p*CH_3_ are C7—H7⋯N1^ii^ (2.60 Å) and C17—H17*A*⋯N1^i^ (2.62 Å). For the sake of comparison to *m*′CN*p*CH_3_, C7—H7⋯Br1^v^ (3.24 Å) and C16—H16*A*⋯Br1^i^ (3.10 Å) in *m*′Br*p*CH_3_, which can be seen in Fig. 7 ▸ as white regions, have distances that are greater than the sum of bromine and hydrogen vdW radii (3.05 Å). Nonetheless, *m*′Br*p*CH_3_ contains a Br⋯Br inter­action, see the red region associated with Br1 in Fig. 7 ▸. This type I halogen bond exhibits a short contact for Br1⋯Br1^iv^ of 3.5565 (5) Å (Cavallo *et al.*, 2016 ▸).

For aromatic rings, π-stacking can exhibit multiple orientations, *e.g.* sandwich, parallel-displaced, and edge-to-face (Wheeler, 2011 ▸), arising largely from dispersion and/or electrostatic inter­actions. The C—H⋯π inter­actions of *m*′CN*p*CH_3_ and *m*′Br*p*CH_3_ resemble the edge-to-face orientation, which is also referred to as a T-shaped orientation. More specifically, the H8⋯3-Ring and H11⋯1-Ring inter­actions of *m*′CN*p*CH_3_ and *m*′Br*p*CH_3_ resemble a bent T-shaped orientation, or the so-called B-T1 orientation as defined by Dinadayalane & Leszczynski (2009 ▸). A computationally derived centroid-to-centroid distance for the B-T1 orientation is 4.63 Å (Dinadayalane & Leszczynski, 2009 ▸), which is close to the centroid distances for the 1-Ring⋯3-Ring^iv^ of *m*′CN*p*CH_3_ (4.60 Å), the 1-Ring⋯3-Ring^iii^ of *m*′Br*p*CH_3_ (4.52 Å), the 3-Ring⋯1-Ring^iii^ of *m*′CN*p*CH_3_ (4.71 Å), and the 3-Ring⋯1-Ring^ii^ of *m*′Br*p*CH_3_ (4.90 Å). See Table 2 ▸ for non-rounded distances and errors.

**Figure 8:**
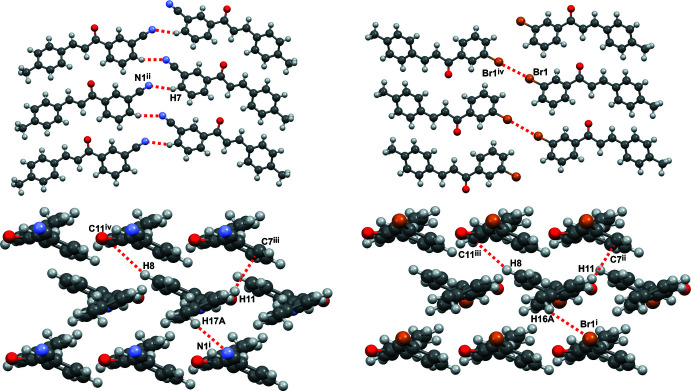
Selected packing displays for *m*′CN*p*CH_3_ (left) and *m*′Br*p*CH_3_ (right) showing identical lateral inter­actions for C16—N1⋯H7 and the Br1⋯Br1 type I halogen bond (top), as well as the stacking inter­actions N1⋯H17*A*, C11⋯H8, C7⋯H11, and Br1⋯H16*A* (bottom). The symmetry codes apply to those mol­ecules inter­acting with the asymmetric unit. Additional N1⋯H7 and Br1⋯Br1 inter­actions are included to serve as a visual aid. Symmetry codes for *m*′CN*p*CH_3_: (i) 1 − *x*, 1 − *y*, 1 − *z*; (ii) −*x*, −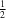 + *y*, 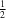 − *z*; (iii) −*x*, 1 − *y*, 1 − *z*; (iv) −*x*, −*y*, 1 - *z.* Symmetry codes for *m*′Br*p*CH_3_: (i) 1 − *x*, 1 − *y*, 1 − *z*; (ii) 1 − *x*, 2 − *y*, 1 − *z*; (iii) −*x*, 2 − *y*, 1 − *z*; (iv) 2 − *x*, 2 − *y*, −*z*.

Inspection of packing diagrams indicate that the *m*′CN*p*CH_3_ mol­ecules form anti­parallel sheets, Fig. 8 ▸. The inter­actions that contribute the most to this stacking are the C—H⋯π inter­actions (C8—H8⋯C11^iv^ or 1-Ring⋯3-Ring^iv^ and C11—H11⋯C7^iii^ or 3-Ring⋯1-Ring^iii^) and C—H⋯N inter­actions (C17—H17*A*⋯N1^i^), Figs. 6 ▸ and 7 ▸. All of these short contacts are less than their respective sum of vdW radii and are expected to contribute to the packing structure. Packing diagrams for *m*′Br*p*CH_3_ also show anti­parallel sheets, Fig. 8 ▸. Similar to *m*′CN*p*CH_3_, the C—H⋯π inter­actions (C8—H8⋯C11^iii^ or 1-Ring⋯3-Ring^iii^ and C11—H11⋯C7^ii^ or 3-Ring⋯1-Ring^ii^) are also contributors to this stacking arrangement. Both chalcones have strong inter­actions that contribute to the lateral arrangement of mol­ecules in the packing diagrams. For *m*′CN*p*CH_3_ this inter­action is the C7—H7⋯N1^i^ inter­action visualized in Fig. 7 ▸. For *m*′Br*p*CH_3_, the Br1⋯Br1^iv^ inter­action, or type 1 halogen bond, contributes to the lateral arrangement.

## Database survey   

A survey of the Cambridge Structural Database (CSD version 5.41, November 2019; Groom *et al.*, 2016 ▸), which excluded chalcones substituted with additional rings, did not yield any mono-substituted cyano­chalcone structures. The only disubstituted cyano­chalcones found contained a *p*CN group on the 3-Ring; 4-cyano-2′-fluoro­chalcone [Bo19p] (LERXOW; *P* 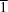; Braun *et al.*, 2006*a* ▸) and 4-cyano-4′-di­ethyl­amino­chalcone [Qp19p] (NAWCEU; *P*2_1_/*c*; Braun *et al.*, 2006*b* ▸). Two of the CN structures, NAWCEU and *m*′CN*p*CH_3_ [Sm6p], share the same space group, *P*2_1_/*c*, while LERXOW belongs to the *P* 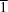 space group. *m*′CN*p*CH_3_ is the first cyano­chalcone crystal structure with a *meta*-cyano substituent and is the first disubstituted cyano-methyl-chalcone structure. Analysis of the close contacts for LERXOW and NAWCEU reveals different inter­actions than for *m*′CN*p*CH_3_. Both structures display no strong inter­actions involving the cyano substituent, and instead both have strong inter­actions involving the carbonyl oxygen and the aromatic hydrogen atoms. LERXOW has a strong inter­action between O1 and H3 and H11, while the oxygen inter­action of note for NAWCEU is between O1 and H14. Additionally, C–H⋯π inter­actions have a lesser impact on the packing structure, as indicated by Hirshfeld analysis. More data are required to assess whether these differences are a function of *meta versus para* cyano substitution.

The same survey, again excluding mol­ecules containing additional rings, showed multiple chalcones containing a bromo substitution, nine of which are substituted in the *meta* position of the 1-Ring, and two of which are disubstituted with a bromo and a methyl group. 3′-Bromo­chalcone [Dm-1] (CICLUW; *P* 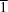; Rosli *et al.*, 2007 ▸) and *m*′Br*p*CH_3_ [Dm6p] belong to the same space group, *P* 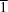. The two disubstituted chalcones most similar to *m*′Br*p*CH_3_, 4′-bromo-4-methyl­chalcone [Dp6p] (IZEFOI; *P*2_1_/*c*; Wang *et al.*, 2004 ▸) and 3-bromo-4′-methyl­chalcone [Fp4m] (IGAPAI; *P* 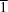; Li *et al.*, 2008 ▸), are the only disubstituted Br/CH_3_ chalcones. Of the two disubstituted chalcones, only IGAPAI shares the same space group as *m*′Br*p*CH_3_, and IGAPAI also exhibits a type I halogen bond (Cavallo *et al.*, 2016 ▸), similar to *m*′Br*p*CH_3_. IZEFOI does display C—H⋯π inter­actions, but these support a parallel arrangement, with the 3-Ring forming close contacts with the 3-Ring of a neighboring mol­ecule, as opposed to the anti­parallel nature of the C—H⋯π inter­actions for *m*′Br*p*CH_3_. *m*′Br*p*CH_3_ is the first methyl-substituted chalcone structure with an *m*′Br atom. Note that the codes Bo19p, Dm-1, Dm6p, Dp6p, Fp4m, Qp19p, and Sm6p are inter­nal codes tied to a large, long-term project.

## Synthesis and crystallization   

**Synthesis.** The preparations of *m*′CN*p*CH_3_ [Sm6p] (Merchant *et al.*, 1965 ▸) and *m*′Br*p*CH_3_ [Dm6p] have previously been reported (Budakoti *et al.*, 2008 ▸; Ellsworth *et al.*, 2008 ▸; Rangarajan *et al.*, 2016 ▸; Soni & Patel, 2017 ▸; Zhang *et al.*, 2017 ▸). Ethanol (1.5 mL, 95%) and a magnetic stir bar were added to two separate Biotage microwave vials (2–5 mL); one contained 4-methyl­benzaldehyde (3 mmol) and the other contained 3′-aceto­phenone (3 mmol). Each vial was heated gently over a hot plate until complete dissolution and then cooled to room temperature; solids may precipitate upon cooling depending on the solubility of the starting material. Once cooled, NaOH (aq) (0.4 mL, 50% by wgt) was added to a benzaldehyde–aceto­phenone mixture. The resulting reaction mixture was vigorously agitated with a microspatula until a slurry formed. Water (2 mL) was added to the vial and its contents were agitated. The vial was capped, centrifuged for one minute, and deca­nted. This trituration was repeated three times. Methanol (2 mL) was added to the vessel and sealed; the microwave-safe vials are safe at high pressures, up to 30 bar. Over a hot plate while stirring, the contents were heated until complete dissolution. Once removed from the heat, the vial was allowed to cool, and crystal growth was observed. Crystals were isolated and dried using vacuum filtration. ^1^H NMR (400 MHz, CDCl_3_, referenced to TMS): δ (ppm) for *m*′Br*p*CH_3_ are 8.13 (*t*, 1H, *J* = 1.7 Hz), 7.93 (*ddd*, 1H, *J* = 7.8, 1.4, 1.0 Hz), 7.80 (*d*, 1H, *J* = 15.6 Hz), 7.70 (*ddd*, 1H, *J* = 8.0, 2.0, 1.0 Hz), 7.55 (*d*, 2H, *J* = 8.1 Hz), 7.40 (*m*, 2H), 7.23 (*d*, 2H, *J* = 8.0 Hz), 2.40 (*s*, 3H); and for *m*′CN*p*CH_3_ are 8.28 (*t*, 1H, *J* = 1.2 Hz), 8.23 (*ddd*, 1H, *J* = 7.9, 1,7, 1.2 Hz), 7.84 (*m*, 2H), 7.64 (*t*, 1H, *J* = 7.9 Hz), 7.56 (*d*, 2H, *J* = 8.1 Hz), 7.43 (*d*, 1H, *J* = 15.6 Hz), 7.25 (*d*, 2H, *J* = 8.5 Hz), 2.41 (*s*, 3H). ^13^C NMR (100 MHz, CDCl_3_, referenced to solvent, 77.16 ppm): δ (ppm) for *m*′Br*p*CH_3_ are 189.25, 145.97, 141.63, 140.29, 135.63, 132.01, 131.60, 130.32, 129.91, 128.77, 127.10, 123.07, 120.51, 21.72; and for *m*′CN*p*CH_3_ are 188.48, 146.82, 142.01, 139.29, 135.65, 132.53, 132.20, 131.73, 129.98, 129.77, 128.87, 119.83, 118.21, 113.20, 21.74.

**Crystallization.** *m*′Br*p*CH_3_ and *m*′CN*p*CH_3_ were crystallized through slow cooling in a Dewar hemispherical low-form flask. Chalcone (20 mg), methanol (0.5 mL), and a magnetic spin vane were added to a conical Biotage microwave vial (0.5–2 mL) and sealed. The tube was placed in boiling water for 1–5 minutes until complete dissolution. While the tube was submerged, two Dewar hemispherical low-form flasks were filled with boiling water and allowed to sit. When the chalcone had nearly dissolved, the Dewar flasks were emptied, and one was placed in a Styrofoam cooler. The Biotage microwave vial was removed from boiling water and placed in the Dewar inside the cooler. The Dewar was filled with boiling water to completely submerge the microwave vial. A round silicone gasket was placed to cover the rim of this Dewar flask before inverting the second Dewar and placing it on top to create a chamber. The cooler was closed with a Styrofoam lid on a low-vibration table in a temperature-regulated room. After 24 h, the vials were removed from the Dewar and crystals were collected using vacuum filtration.

## Refinement   

Crystal data, data collection and structure refinement details are summarized in Table 3 ▸. The X-ray intensity data for each chalcone derivative was measured at 100 K on a Bruker Photon II D8 Venture diffractometer equipped with both IμS-Cu and IμS-Mo microfocus X-ray sources. The Cu *K*α (λ = 1.54178 Å) source was used for all crystallographic investigations. Data sets were corrected for Lorentz and polarization effects as well as absorption. The criterion for observed reflections is *I* > 2σ(*I*). Lattice parameters were determined from least-squares analysis of reflection data. Empirical absorption corrections were applied using *SADABS* (Krause *et al.*, 2015 ▸). Structures were solved by direct methods and refined by full-matrix least-squares analysis on *F* ^2^ using *X-SEED* equipped with *SHELXT* (Barbour, 2001 ▸ and Sheldrick, 2015*a* ▸). All non-hydrogen atoms were refined anisotropically by full-matrix least-squares on *F* ^2^ using the *SHELXL* program (Sheldrick, 2015*b* ▸). H atoms (for OH and NH) were located in a difference-Fourier synthesis and refined isotropically with independent O/N—H distances or restrained to 0.85 (2) Å. The remaining H atoms were included in idealized geometric positions with *U*_iso_(H) = 1.2*U*_eq_(parent atom) or 1.5*U*_eq_(C-meth­yl).

Unit cells were visualized with *Mercury 2020.1* (Macrae *et al.*, 2020 ▸), Hirshfeld analyses were executed with *Crystal Explorer 17.5* (Turner *et al.*, 2017 ▸), while distance/angle measurements as well as *ORTEP* images were captured using *OLEX2* (Dolomanov *et al.*, 2009 ▸).

## Supplementary Material

Crystal structure: contains datablock(s) I, II. DOI: 10.1107/S2056989020011135/tx2030sup1.cif

Structure factors: contains datablock(s) I. DOI: 10.1107/S2056989020011135/tx2030Isup2.hkl

Structure factors: contains datablock(s) II. DOI: 10.1107/S2056989020011135/tx2030IIsup3.hkl

Hirshfeld fingerprint plots. DOI: 10.1107/S2056989020011135/tx2030sup4.pdf

Supporting information file. DOI: 10.1107/S2056989020011135/tx2030Isup5.cml

Supporting information file. DOI: 10.1107/S2056989020011135/tx2030IIsup6.cml

CCDC references: 2023082, 2023081

Additional supporting information:  crystallographic information; 3D view; checkCIF report

## Figures and Tables

**Figure 2:**
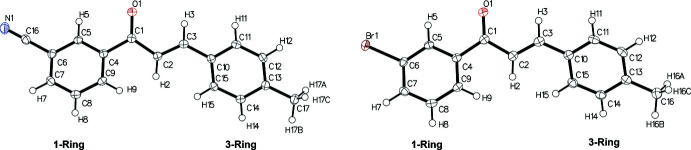
The asymmetric units of *m*′CN*p*CH_3_ (left) and *m*′Br*p*CH_3_ (right) showing the atom labeling with displacement ellipsoids drawn at the 50% probability level.

**Table 1:** Selected angles (°) The Φ1, Φ2, and Φ3 dihedrals are defined by C5—C4—C1—C2, C4—C1—C2—C3, and C2—C3—C10—C11, respectively.

Chalcone	Φ1	Φ2	Φ3	1-Ring 3-Ring Twist	1-Ring 3-Ring Fold
*m*′CN*p*CH_3_	−154.58 (10)	−169.15 (10)	−163.34 (10)	49.11 (4)	0.67 (4)
*m*′Br*p*CH_3_	−153.51 (16)	−169.73 (17)	−161.99 (18)	49.15 (6)	1.55 (6)

**Table 2:** Distances (Å) for close contacts Distances to the 1-Ring and 3-Ring reflect the distances to the centroids of those rings. The sums of the van der Waals radii (Å) for hydrogen plus carbon, nitro­gen, or bromine are 2.9, 2.75, and 3.05, respectively, while the sum for bromine plus bromine is 3.7. The symmetry codes apply to those mol­ecules inter­acting with the asymmetric unit. Estimated standard deviations are listed in parentheses.

*m*′CN*p*CH_3_	Distance	*m*′Br*p*CH_3_	Distance
C8—H8⋯C11^iv^	2.8835 (11)	C8—H8⋯C11^iii^	2.8019 (16)
C8—H8⋯3-Ring^iv^	2.7391 (4)	C8—H8⋯3-Ring^iii^	2.709 (2)
C11—H11⋯C7^iii^	2.8187 (11)	C11—H11⋯C7^ii^	2.8936 (17)
C11—H11⋯1-Ring^iii^	2.8866 (4)	C11—H11⋯1-Ring^ii^	3.061 (2)
1-Ring⋯3-Ring^iv^	4.6036 (6)	1-Ring⋯3-Ring^iii^	4.5176 (10)
3-Ring⋯1-Ring^iii^	4.7132 (6)	3-Ring⋯1-Ring^ii^	4.8989 (11)
C7—H7⋯N1^ii^	2.5999 (9)	C7—H7⋯Br1^v^	3.2379 (4)
C17—H17*A*⋯N1^i^	2.6168 (11)	C16—H16*A*⋯Br1^i^	3.0962 (3)
		Br1⋯Br1^iv^	3.5556 (5)

Symmetry codes for *m*′CN*p*CH_3_: (i) 1 − *x*, 1 − *y*, 1 − *z*; (ii) −*x*, −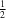 + *y*, 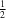 − *z*; (iii) −*x*, 1 − *y*, 1 − *z*; (iv) −*x*, −*y*, 1 − *z*. Symmetry codes for *m*′Br*p*CH_3_: (i) 1 − *x*, 1 − *y*, 1 − *z*; (ii) 1 − *x*, 2 − *y*, 1 − *z*; (iii) −*x*, 2 − *y*, 1 − *z*; (iv) 2 − *x*, 2 − *y*, −*z*; (v) 1 − *x*, 2 − *y*, −*z*.

**Table 3:** Experimental details

	*m*′CN*p*CH_3_	*m*′Br*p*CH_3_
Crystal data		
Chemical formula	C_17_H_13_NO	C_16_H_13_BrO
*M*_r_	247.28	301.17
Crystal system, space group	Monoclinic, *P*2_1_/*c*	Triclinic, *P* 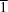
Temperature (K)	100	100
*a*, *b*, *c* (Å)	7.2986 (1), 5.8504 (1), 29.7783 (5)	5.9282 (6), 7.3614 (8), 14.6747 (16)
α, β, γ (°)	90, 94.525 (1), 90	88.532 (3), 82.199 (3), 87.457 (3)
*V* (Å^3^)	1267.56 (4)	633.73 (12)
*Z*	4	2
*D* *_x_* (Mg m^−3^)	1.296	1.578
Radiation type	Cu *K*α	Cu *K*α
μ (mm^−1^)	0.64	4.28
Crystal shape	Transparent plate	Transparent plate
Colour	Colourless	Colorless
Crystal size (mm)	0.35 × 0.21 × 0.09	0.39 × 0.25 × 0.11

Data collection		
Diffractometer	Bruker D8 Venture	Bruker D8 Venture
Absorption correction	Multi-scan (*SADABS*; Krause *et al.*, 2015 ▸)	Multi-scan (*SADABS*; Krause *et al.*, 2015 ▸)
*T*_min_, *T*_max_	0.676, 0.754	0.531, 0.754
No. of measured, independent and observed [*I* > 2σ(*I*)] reflections	13801, 2494, 2213	8397, 2461, 2440
*R*_int_	0.028	0.023
(sin θ/λ)_max_ (Å^−1^)	0.617	0.617

Refinement		
*R*[*F* ^2^ > 2σ(*F* ^2^)], *wR*(*F* ^2^), *S*	0.033, 0.087, 1.03	0.026, 0.065, 1.09
No. of reflections	2494	2461
No. of parameters	173	164
H-atom treatment	H-atom parameters constrained	H-atom parameters constrained
Δρ_max_, Δρ_min_ (e Å^−3^)	0.21, −0.18	0.63, −0.40

Computer programs: *APEX3* (Bruker, 2018 ▸), *SAINT* (Bruker, 2017 ▸), *SHELXT2018/2* (Sheldrick, 2015*a* ▸), *SHELXL2018/3* (Sheldrick, 2015*b* ▸) and *X-SEED* (Barbour, 2001 ▸).

## supplementary crystallographic information

### 2-[3-(4-Methylphenyl)prop-2-enoyl]benzonitrile (I) Crystal data

**Table:**

C_17_H_13_NO	*F*(000) = 520
*M**_r_* = 247.28	*D*_x_ = 1.296 Mg m^−^^3^
Monoclinic, *P*2_1_/*c*	Cu *K*α radiation, λ = 1.54178 Å
*a* = 7.2986 (1) Å	Cell parameters from 7537 reflections
*b* = 5.8504 (1) Å	θ = 3.0–72.1°
*c* = 29.7783 (5) Å	µ = 0.64 mm^−^^1^
β = 94.525 (1)°	*T* = 100 K
*V* = 1267.56 (4) Å^3^	Transparent plate, colourless
*Z* = 4	0.35 × 0.21 × 0.09 mm

### 2-[3-(4-Methylphenyl)prop-2-enoyl]benzonitrile (I) Data collection

**Table:**

Bruker D8 Venture diffractometer	2494 independent reflections
Radiation source: Microsource IuS Incoatec 3.0	2213 reflections with *I* > 2σ(*I*)
Double Bounce Multilayer Mirrors monochromator	*R*_int_ = 0.028
Detector resolution: 7.9 pixels mm^-1^	θ_max_ = 72.1°, θ_min_ = 3.0°
φ and ω scans	*h* = −9→8
Absorption correction: multi-scan (SADABS; Krause *et al.*, 2015)	*k* = −7→7
*T*_min_ = 0.676, *T*_max_ = 0.754	*l* = −36→36
13801 measured reflections	

### 2-[3-(4-Methylphenyl)prop-2-enoyl]benzonitrile (I) Refinement

**Table:**

Refinement on *F*^2^	Primary atom site location: structure-invariant direct methods
Least-squares matrix: full	Hydrogen site location: inferred from neighbouring sites
*R*[*F*^2^ > 2σ(*F*^2^)] = 0.033	H-atom parameters constrained
*wR*(*F*^2^) = 0.087	*w* = 1/[σ^2^(*F*_o_^2^) + (0.045*P*)^2^ + 0.4265*P*] where *P* = (*F*_o_^2^ + 2*F*_c_^2^)/3
*S* = 1.03	(Δ/σ)_max_ = 0.001
2494 reflections	Δρ_max_ = 0.21 e Å^−^^3^
173 parameters	Δρ_min_ = −0.18 e Å^−^^3^
0 restraints	

### 2-[3-(4-Methylphenyl)prop-2-enoyl]benzonitrile (I) Special details

**Table:**

Geometry. All esds (except the esd in the dihedral angle between two l.s. planes) are estimated using the full covariance matrix. The cell esds are taken into account individually in the estimation of esds in distances, angles and torsion angles; correlations between esds in cell parameters are only used when they are defined by crystal symmetry. An approximate (isotropic) treatment of cell esds is used for estimating esds involving l.s. planes.
Refinement. All nonhydrogen atoms were located in a single difference Fourier electron density map and refined using anisotropic displacement parameters. All C-H hydrogen atoms were placed in calculated positions with Uiso = 1.2xUeqiv of the connected C atoms (1.5xUeqiv for methyl groups).

### 2-[3-(4-Methylphenyl)prop-2-enoyl]benzonitrile (I) Fractional atomic coordinates and isotropic or equivalent isotropic displacement parameters (Å^2^)

**Table:**

	*x*	*y*	*z*	*U*_iso_*/*U*_eq_	
O1	0.22192 (11)	0.72690 (13)	0.47783 (2)	0.02168 (19)	
N1	0.13188 (15)	0.75910 (19)	0.27529 (3)	0.0286 (2)	
C1	0.21471 (14)	0.52052 (18)	0.47083 (3)	0.0165 (2)	
C2	0.26906 (14)	0.35011 (18)	0.50599 (3)	0.0174 (2)	
H2	0.282576	0.194303	0.497894	0.021*	
C3	0.29943 (14)	0.41308 (18)	0.54913 (3)	0.0160 (2)	
H3	0.282067	0.570173	0.555670	0.019*	
C4	0.14995 (14)	0.43210 (18)	0.42488 (3)	0.0159 (2)	
C5	0.16826 (14)	0.57332 (18)	0.38769 (3)	0.0163 (2)	
H5	0.221501	0.720872	0.391577	0.020*	
C6	0.10757 (14)	0.49562 (19)	0.34474 (3)	0.0171 (2)	
C7	0.02732 (14)	0.27968 (19)	0.33848 (4)	0.0187 (2)	
H7	−0.013000	0.227830	0.309115	0.022*	
C8	0.00746 (14)	0.14251 (19)	0.37570 (4)	0.0189 (2)	
H8	−0.049184	−0.003134	0.371874	0.023*	
C9	0.06980 (14)	0.21624 (18)	0.41866 (4)	0.0176 (2)	
H9	0.057822	0.119396	0.443864	0.021*	
C10	0.35662 (14)	0.26495 (18)	0.58726 (3)	0.0152 (2)	
C11	0.33577 (14)	0.34331 (19)	0.63106 (3)	0.0173 (2)	
H11	0.287002	0.491628	0.635291	0.021*	
C12	0.38509 (15)	0.20805 (19)	0.66834 (3)	0.0186 (2)	
H12	0.366765	0.263493	0.697639	0.022*	
C13	0.46133 (14)	−0.00854 (19)	0.66336 (3)	0.0173 (2)	
C14	0.48597 (14)	−0.08447 (18)	0.61969 (4)	0.0171 (2)	
H14	0.540026	−0.229971	0.615618	0.021*	
C15	0.43333 (14)	0.04771 (18)	0.58221 (3)	0.0164 (2)	
H15	0.449373	−0.009384	0.552915	0.020*	
C16	0.12291 (15)	0.6427 (2)	0.30610 (4)	0.0206 (2)	
C17	0.51598 (16)	−0.1542 (2)	0.70393 (4)	0.0227 (2)	
H17A	0.627558	−0.091602	0.719826	0.034*	
H17B	0.539551	−0.310760	0.694210	0.034*	
H17C	0.416382	−0.154959	0.724164	0.034*	

### 2-[3-(4-Methylphenyl)prop-2-enoyl]benzonitrile (I) Atomic displacement parameters (Å^2^)

**Table:**

	*U*^11^	*U*^22^	*U*^33^	*U*^12^	*U*^13^	*U*^23^
O1	0.0290 (4)	0.0159 (4)	0.0197 (4)	0.0010 (3)	−0.0009 (3)	−0.0006 (3)
N1	0.0352 (6)	0.0302 (6)	0.0197 (5)	−0.0055 (5)	−0.0016 (4)	0.0035 (4)
C1	0.0144 (5)	0.0176 (5)	0.0178 (5)	0.0007 (4)	0.0024 (4)	−0.0003 (4)
C2	0.0185 (5)	0.0157 (5)	0.0179 (5)	0.0019 (4)	0.0012 (4)	0.0001 (4)
C3	0.0137 (5)	0.0149 (5)	0.0196 (5)	−0.0004 (4)	0.0019 (4)	−0.0003 (4)
C4	0.0139 (5)	0.0163 (5)	0.0174 (5)	0.0035 (4)	0.0011 (4)	0.0000 (4)
C5	0.0146 (5)	0.0149 (5)	0.0192 (5)	0.0016 (4)	0.0007 (4)	0.0000 (4)
C6	0.0155 (5)	0.0186 (5)	0.0172 (5)	0.0031 (4)	0.0010 (4)	0.0015 (4)
C7	0.0171 (5)	0.0205 (5)	0.0182 (5)	0.0033 (4)	−0.0013 (4)	−0.0039 (4)
C8	0.0168 (5)	0.0152 (5)	0.0244 (5)	0.0006 (4)	−0.0003 (4)	−0.0013 (4)
C9	0.0164 (5)	0.0162 (5)	0.0202 (5)	0.0022 (4)	0.0014 (4)	0.0023 (4)
C10	0.0128 (5)	0.0164 (5)	0.0161 (5)	−0.0023 (4)	0.0005 (4)	−0.0005 (4)
C11	0.0167 (5)	0.0156 (5)	0.0195 (5)	−0.0010 (4)	0.0018 (4)	−0.0023 (4)
C12	0.0205 (5)	0.0209 (5)	0.0146 (5)	−0.0024 (4)	0.0017 (4)	−0.0034 (4)
C13	0.0160 (5)	0.0193 (5)	0.0164 (5)	−0.0036 (4)	−0.0004 (4)	0.0015 (4)
C14	0.0162 (5)	0.0148 (5)	0.0205 (5)	−0.0001 (4)	0.0020 (4)	−0.0004 (4)
C15	0.0158 (5)	0.0185 (5)	0.0150 (5)	−0.0010 (4)	0.0021 (4)	−0.0014 (4)
C16	0.0210 (5)	0.0222 (6)	0.0182 (5)	−0.0011 (4)	−0.0013 (4)	−0.0024 (5)
C17	0.0260 (6)	0.0230 (6)	0.0188 (5)	−0.0008 (5)	−0.0001 (4)	0.0039 (4)

### 2-[3-(4-Methylphenyl)prop-2-enoyl]benzonitrile (I) Geometric parameters (Å, º)

**Table:**

O1—C1	1.2257 (13)	C8—H8	0.9500
N1—C16	1.1487 (15)	C9—H9	0.9500
C1—C2	1.4771 (15)	C10—C15	1.4017 (15)
C1—C4	1.5038 (14)	C10—C11	1.4021 (14)
C2—C3	1.3380 (15)	C11—C12	1.3875 (15)
C2—H2	0.9500	C11—H11	0.9500
C3—C10	1.4629 (14)	C12—C13	1.3965 (16)
C3—H3	0.9500	C12—H12	0.9500
C4—C5	1.3966 (15)	C13—C14	1.3992 (15)
C4—C9	1.3980 (15)	C13—C17	1.5062 (14)
C5—C6	1.3961 (14)	C14—C15	1.3869 (15)
C5—H5	0.9500	C14—H14	0.9500
C6—C7	1.3988 (16)	C15—H15	0.9500
C6—C16	1.4482 (15)	C17—H17A	0.9800
C7—C8	1.3852 (16)	C17—H17B	0.9800
C7—H7	0.9500	C17—H17C	0.9800
C8—C9	1.3920 (15)		
			
O1—C1—C2	122.61 (10)	C4—C9—H9	119.8
O1—C1—C4	119.98 (9)	C15—C10—C11	118.03 (9)
C2—C1—C4	117.41 (9)	C15—C10—C3	123.08 (9)
C3—C2—C1	120.63 (10)	C11—C10—C3	118.89 (10)
C3—C2—H2	119.7	C12—C11—C10	121.16 (10)
C1—C2—H2	119.7	C12—C11—H11	119.4
C2—C3—C10	126.70 (10)	C10—C11—H11	119.4
C2—C3—H3	116.6	C11—C12—C13	120.86 (10)
C10—C3—H3	116.6	C11—C12—H12	119.6
C5—C4—C9	119.63 (10)	C13—C12—H12	119.6
C5—C4—C1	118.38 (10)	C12—C13—C14	117.92 (9)
C9—C4—C1	121.98 (9)	C12—C13—C17	120.70 (9)
C6—C5—C4	119.38 (10)	C14—C13—C17	121.37 (10)
C6—C5—H5	120.3	C15—C14—C13	121.54 (10)
C4—C5—H5	120.3	C15—C14—H14	119.2
C5—C6—C7	121.03 (10)	C13—C14—H14	119.2
C5—C6—C16	119.71 (10)	C14—C15—C10	120.45 (10)
C7—C6—C16	119.24 (9)	C14—C15—H15	119.8
C8—C7—C6	119.06 (10)	C10—C15—H15	119.8
C8—C7—H7	120.5	N1—C16—C6	178.83 (12)
C6—C7—H7	120.5	C13—C17—H17A	109.5
C7—C8—C9	120.57 (10)	C13—C17—H17B	109.5
C7—C8—H8	119.7	H17A—C17—H17B	109.5
C9—C8—H8	119.7	C13—C17—H17C	109.5
C8—C9—C4	120.32 (10)	H17A—C17—H17C	109.5
C8—C9—H9	119.8	H17B—C17—H17C	109.5
			
O1—C1—C2—C3	11.34 (17)	C5—C4—C9—C8	−0.38 (15)
C4—C1—C2—C3	−169.15 (10)	C1—C4—C9—C8	178.43 (9)
C1—C2—C3—C10	−178.91 (9)	C2—C3—C10—C15	17.24 (17)
O1—C1—C4—C5	24.95 (15)	C2—C3—C10—C11	−163.34 (10)
C2—C1—C4—C5	−154.58 (10)	C15—C10—C11—C12	−1.61 (15)
O1—C1—C4—C9	−153.88 (10)	C3—C10—C11—C12	178.95 (9)
C2—C1—C4—C9	26.59 (14)	C10—C11—C12—C13	1.57 (16)
C9—C4—C5—C6	−0.58 (15)	C11—C12—C13—C14	−0.10 (16)
C1—C4—C5—C6	−179.43 (9)	C11—C12—C13—C17	179.55 (10)
C4—C5—C6—C7	0.60 (16)	C12—C13—C14—C15	−1.32 (16)
C4—C5—C6—C16	178.85 (9)	C17—C13—C14—C15	179.03 (10)
C5—C6—C7—C8	0.34 (16)	C13—C14—C15—C10	1.28 (16)
C16—C6—C7—C8	−177.92 (10)	C11—C10—C15—C14	0.19 (15)
C6—C7—C8—C9	−1.30 (16)	C3—C10—C15—C14	179.62 (10)
C7—C8—C9—C4	1.34 (16)		

### 1-(2-Bromophenyl)-3-(4-methylphenyl)prop-2-en-1-one (II) Crystal data

**Table:**

C_16_H_13_BrO	*Z* = 2
*M**_r_* = 301.17	*F*(000) = 304
Triclinic, *P*1	*D*_x_ = 1.578 Mg m^−^^3^
*a* = 5.9282 (6) Å	Cu *K*α radiation, λ = 1.54178 Å
*b* = 7.3614 (8) Å	Cell parameters from 1318 reflections
*c* = 14.6747 (16) Å	θ = 6.0–71.7°
α = 88.532 (3)°	µ = 4.28 mm^−^^1^
β = 82.199 (3)°	*T* = 100 K
γ = 87.457 (3)°	Transparent plate, colorless
*V* = 633.73 (12) Å^3^	0.39 × 0.25 × 0.11 mm

### 1-(2-Bromophenyl)-3-(4-methylphenyl)prop-2-en-1-one (II) Data collection

**Table:**

Bruker D8 Venture diffractometer	2461 independent reflections
Radiation source: Microsource IuS Incoatec 3.0	2440 reflections with *I* > 2σ(*I*)
Double Bounce Multilayer Mirrors monochromator	*R*_int_ = 0.023
Detector resolution: 7.9 pixels mm^-1^	θ_max_ = 72.2°, θ_min_ = 3.0°
φ and ω scans	*h* = −6→7
Absorption correction: multi-scan (SADABS; Krause *et al.*, 2015)	*k* = −9→9
*T*_min_ = 0.531, *T*_max_ = 0.754	*l* = −18→18
8397 measured reflections	

### 1-(2-Bromophenyl)-3-(4-methylphenyl)prop-2-en-1-one (II) Refinement

**Table:**

Refinement on *F*^2^	Primary atom site location: structure-invariant direct methods
Least-squares matrix: full	Hydrogen site location: inferred from neighbouring sites
*R*[*F*^2^ > 2σ(*F*^2^)] = 0.026	H-atom parameters constrained
*wR*(*F*^2^) = 0.065	*w* = 1/[σ^2^(*F*_o_^2^) + (0.0313*P*)^2^ + 0.5783*P*] where *P* = (*F*_o_^2^ + 2*F*_c_^2^)/3
*S* = 1.09	(Δ/σ)_max_ = 0.001
2461 reflections	Δρ_max_ = 0.63 e Å^−^^3^
164 parameters	Δρ_min_ = −0.40 e Å^−^^3^
0 restraints	

### 1-(2-Bromophenyl)-3-(4-methylphenyl)prop-2-en-1-one (II) Special details

**Table:**

Geometry. All esds (except the esd in the dihedral angle between two l.s. planes) are estimated using the full covariance matrix. The cell esds are taken into account individually in the estimation of esds in distances, angles and torsion angles; correlations between esds in cell parameters are only used when they are defined by crystal symmetry. An approximate (isotropic) treatment of cell esds is used for estimating esds involving l.s. planes.
Refinement. All nonhydrogen atoms were located in a single difference Fourier electron density map and refined using anisotropic displacement parameters. All C-H hydrogen atoms were placed in calculated positions with Uiso = 1.2xUeqiv of the connected C atoms (1.5xUeqiv for methyl groups).

### 1-(2-Bromophenyl)-3-(4-methylphenyl)prop-2-en-1-one (II) Fractional atomic coordinates and isotropic or equivalent isotropic displacement parameters (Å^2^)

**Table:**

	*x*	*y*	*z*	*U*_iso_*/*U*_eq_	
Br1	0.80035 (3)	0.89029 (3)	0.08237 (2)	0.02192 (9)	
O1	0.7329 (2)	0.7890 (2)	0.45698 (9)	0.0223 (3)	
C1	0.5354 (3)	0.7917 (2)	0.44225 (13)	0.0170 (4)	
C2	0.3466 (3)	0.7319 (3)	0.51279 (13)	0.0192 (4)	
H2	0.199245	0.718770	0.495759	0.023*	
C3	0.3817 (3)	0.6965 (2)	0.59951 (13)	0.0186 (4)	
H3	0.530796	0.713033	0.613915	0.022*	
C4	0.4726 (3)	0.8590 (2)	0.35130 (13)	0.0155 (3)	
C5	0.6366 (3)	0.8439 (2)	0.27330 (13)	0.0161 (3)	
H5	0.783980	0.791310	0.277957	0.019*	
C6	0.5803 (3)	0.9069 (2)	0.18959 (12)	0.0167 (3)	
C7	0.3658 (3)	0.9867 (2)	0.18070 (13)	0.0189 (4)	
H7	0.330243	1.029208	0.122449	0.023*	
C8	0.2067 (3)	1.0024 (2)	0.25840 (14)	0.0192 (4)	
H8	0.061052	1.058189	0.253582	0.023*	
C9	0.2567 (3)	0.9378 (2)	0.34359 (13)	0.0175 (4)	
H9	0.144730	0.947060	0.396286	0.021*	
C10	0.2121 (3)	0.6346 (2)	0.67464 (13)	0.0180 (4)	
C11	0.2535 (3)	0.6525 (2)	0.76554 (14)	0.0197 (4)	
H11	0.393474	0.699115	0.777189	0.024*	
C12	0.0940 (3)	0.6036 (3)	0.83870 (13)	0.0204 (4)	
H12	0.124142	0.620360	0.899810	0.025*	
C13	−0.1104 (3)	0.5301 (2)	0.82409 (13)	0.0188 (4)	
C14	−0.1492 (3)	0.5068 (2)	0.73322 (13)	0.0191 (4)	
H14	−0.285712	0.453991	0.721786	0.023*	
C15	0.0076 (3)	0.5594 (3)	0.65972 (13)	0.0195 (4)	
H15	−0.023652	0.544247	0.598635	0.023*	
C16	−0.2846 (4)	0.4761 (3)	0.90371 (14)	0.0243 (4)	
H16A	−0.238535	0.357702	0.928866	0.036*	
H16B	−0.433393	0.467934	0.882360	0.036*	
H16C	−0.295102	0.567581	0.951623	0.036*	

### 1-(2-Bromophenyl)-3-(4-methylphenyl)prop-2-en-1-one (II) Atomic displacement parameters (Å^2^)

**Table:**

	*U*^11^	*U*^22^	*U*^33^	*U*^12^	*U*^13^	*U*^23^
Br1	0.02062 (13)	0.02741 (13)	0.01669 (12)	−0.00258 (8)	0.00161 (8)	0.00044 (8)
O1	0.0152 (7)	0.0321 (7)	0.0196 (7)	−0.0023 (5)	−0.0024 (5)	0.0007 (5)
C1	0.0174 (9)	0.0142 (8)	0.0194 (9)	−0.0018 (7)	−0.0019 (7)	−0.0021 (6)
C2	0.0175 (9)	0.0191 (9)	0.0206 (9)	−0.0019 (7)	−0.0008 (7)	−0.0013 (7)
C3	0.0176 (9)	0.0142 (8)	0.0237 (9)	0.0005 (7)	−0.0015 (7)	−0.0026 (7)
C4	0.0150 (8)	0.0131 (8)	0.0188 (9)	−0.0030 (6)	−0.0025 (7)	−0.0012 (6)
C5	0.0136 (8)	0.0143 (8)	0.0205 (9)	−0.0026 (6)	−0.0024 (7)	−0.0016 (7)
C6	0.0163 (9)	0.0167 (8)	0.0168 (8)	−0.0043 (7)	0.0005 (7)	−0.0012 (6)
C7	0.0198 (9)	0.0174 (8)	0.0208 (9)	−0.0042 (7)	−0.0070 (7)	0.0020 (7)
C8	0.0140 (9)	0.0167 (8)	0.0275 (10)	−0.0003 (7)	−0.0048 (7)	−0.0008 (7)
C9	0.0147 (9)	0.0157 (8)	0.0215 (9)	−0.0018 (7)	0.0001 (7)	−0.0021 (7)
C10	0.0183 (9)	0.0148 (8)	0.0209 (9)	0.0002 (7)	−0.0026 (7)	0.0011 (7)
C11	0.0196 (9)	0.0155 (8)	0.0259 (10)	−0.0007 (7)	−0.0099 (8)	0.0010 (7)
C12	0.0245 (10)	0.0175 (9)	0.0207 (9)	0.0020 (7)	−0.0092 (8)	0.0001 (7)
C13	0.0206 (9)	0.0142 (8)	0.0218 (9)	0.0030 (7)	−0.0045 (7)	0.0000 (7)
C14	0.0187 (9)	0.0167 (8)	0.0230 (9)	−0.0025 (7)	−0.0058 (7)	−0.0003 (7)
C15	0.0217 (10)	0.0189 (9)	0.0189 (9)	−0.0027 (7)	−0.0054 (7)	−0.0012 (7)
C16	0.0244 (10)	0.0272 (10)	0.0211 (9)	0.0001 (8)	−0.0027 (8)	−0.0006 (8)

### 1-(2-Bromophenyl)-3-(4-methylphenyl)prop-2-en-1-one (II) Geometric parameters (Å, º)

**Table:**

Br1—C6	1.9053 (18)	C8—H8	0.9500
O1—C1	1.219 (2)	C9—H9	0.9500
C1—C2	1.491 (3)	C10—C11	1.399 (3)
C1—C4	1.500 (3)	C10—C15	1.401 (3)
C2—C3	1.334 (3)	C11—C12	1.382 (3)
C2—H2	0.9500	C11—H11	0.9500
C3—C10	1.465 (3)	C12—C13	1.393 (3)
C3—H3	0.9500	C12—H12	0.9500
C4—C9	1.399 (3)	C13—C14	1.400 (3)
C4—C5	1.401 (3)	C13—C16	1.508 (3)
C5—C6	1.380 (3)	C14—C15	1.384 (3)
C5—H5	0.9500	C14—H14	0.9500
C6—C7	1.398 (3)	C15—H15	0.9500
C7—C8	1.382 (3)	C16—H16A	0.9800
C7—H7	0.9500	C16—H16B	0.9800
C8—C9	1.391 (3)	C16—H16C	0.9800
			
O1—C1—C2	122.32 (17)	C4—C9—H9	120.2
O1—C1—C4	120.48 (17)	C11—C10—C15	118.05 (18)
C2—C1—C4	117.20 (16)	C11—C10—C3	119.08 (17)
C3—C2—C1	120.92 (17)	C15—C10—C3	122.87 (17)
C3—C2—H2	119.5	C12—C11—C10	121.11 (18)
C1—C2—H2	119.5	C12—C11—H11	119.4
C2—C3—C10	126.21 (18)	C10—C11—H11	119.4
C2—C3—H3	116.9	C11—C12—C13	120.94 (18)
C10—C3—H3	116.9	C11—C12—H12	119.5
C9—C4—C5	120.03 (17)	C13—C12—H12	119.5
C9—C4—C1	121.39 (17)	C12—C13—C14	118.08 (18)
C5—C4—C1	118.57 (16)	C12—C13—C16	121.12 (18)
C6—C5—C4	118.80 (17)	C14—C13—C16	120.80 (18)
C6—C5—H5	120.6	C15—C14—C13	121.21 (18)
C4—C5—H5	120.6	C15—C14—H14	119.4
C5—C6—C7	121.94 (17)	C13—C14—H14	119.4
C5—C6—Br1	119.84 (14)	C14—C15—C10	120.57 (18)
C7—C6—Br1	118.21 (14)	C14—C15—H15	119.7
C8—C7—C6	118.60 (17)	C10—C15—H15	119.7
C8—C7—H7	120.7	C13—C16—H16A	109.5
C6—C7—H7	120.7	C13—C16—H16B	109.5
C7—C8—C9	120.93 (17)	H16A—C16—H16B	109.5
C7—C8—H8	119.5	C13—C16—H16C	109.5
C9—C8—H8	119.5	H16A—C16—H16C	109.5
C8—C9—C4	119.69 (17)	H16B—C16—H16C	109.5
C8—C9—H9	120.2		
			
O1—C1—C2—C3	9.4 (3)	C5—C4—C9—C8	−0.6 (3)
C4—C1—C2—C3	−169.73 (17)	C1—C4—C9—C8	178.53 (16)
C1—C2—C3—C10	−179.08 (17)	C2—C3—C10—C11	−161.99 (18)
O1—C1—C4—C9	−151.81 (18)	C2—C3—C10—C15	17.3 (3)
C2—C1—C4—C9	27.3 (2)	C15—C10—C11—C12	−2.3 (3)
O1—C1—C4—C5	27.4 (3)	C3—C10—C11—C12	177.07 (16)
C2—C1—C4—C5	−153.51 (16)	C10—C11—C12—C13	1.8 (3)
C9—C4—C5—C6	−0.4 (3)	C11—C12—C13—C14	0.2 (3)
C1—C4—C5—C6	−179.54 (16)	C11—C12—C13—C16	179.84 (17)
C4—C5—C6—C7	0.7 (3)	C12—C13—C14—C15	−1.6 (3)
C4—C5—C6—Br1	179.46 (13)	C16—C13—C14—C15	178.69 (17)
C5—C6—C7—C8	0.0 (3)	C13—C14—C15—C10	1.1 (3)
Br1—C6—C7—C8	−178.79 (13)	C11—C10—C15—C14	0.8 (3)
C6—C7—C8—C9	−1.0 (3)	C3—C10—C15—C14	−178.51 (17)
C7—C8—C9—C4	1.3 (3)		
