# Supplementary material for: Crystal structure and Hirshfeld analysis of 3′-bromo-4-methyl­chalcone and 3′-cyano-4-methyl­chalcone
Source: Acta Crystallogr E Crystallogr Commun. 2020 Aug 25;76(Pt 9):1496–502. doi: 10.1107/S2056989020011135 (PMC7472750; doi:10.1107/S2056989020011135)
Supplement: Supplementary file 4 [file e-76-01496-sup4.pdf]

Supporting Information

Hirshfeld Fingerprint Plots:

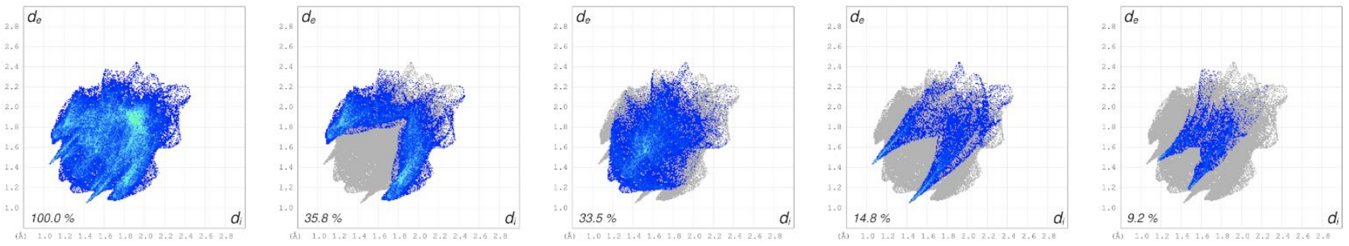

SI Figure 1. The two-dimensional fingerprint plots showing the percentage of contacts found by Hirshfeld surface analysis of *m'*CN*p*CH_3. The contacts are shown from left to right: all, H⋯C (35.8%), H⋯H (33.5%), H⋯N (14.8%), and H⋯O (9.2%). These four contacts make up 93.3 % of the total. Not pictured are the fingerprint plots showing the less prominent C⋯C (4.1%), C⋯O (1.9%), N⋯N (0.5%), N⋯C (0.3%) contacts. For reference, the percentage contributions of the *m*'CN*p*CH_3 intermolecular contacts to the total Hirshfeld surface are: C⋯H/H⋯C (35.8%), H⋯H (33.5%), N⋯H/H⋯N (14.8%), O⋯H/H⋯O (9.2%), C⋯C (4.1%), O⋯C/C⋯O (1.9%), N⋯N (0.5%), C⋯N/N⋯C (0.3%).

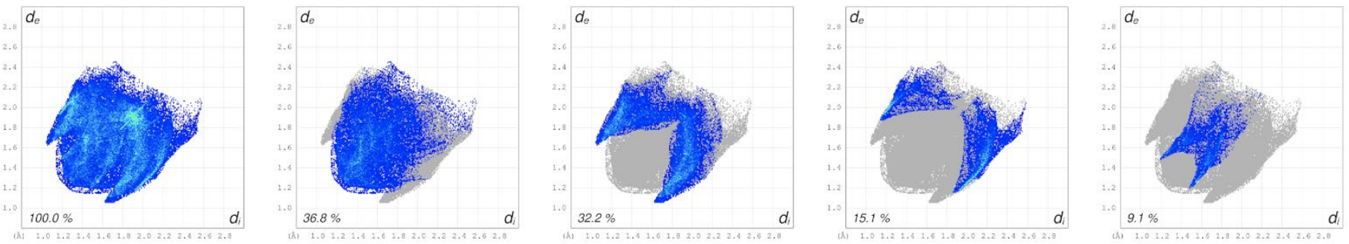

SI Figure 2. The two-dimensional fingerprint plots showing the percentage of contacts found by Hirshfeld surface analysis of *m'*CN*p*CH_3. The contacts are shown from left to right, all, H⋯H (36.8%), H⋯C (32.2%), H⋯Br (15.1%), and H⋯O (9.1%). These four contacts make up 93.2 % of the total. Not pictured are the fingerprint plots showing the less prominent C⋯C (3.4%), C⋯O (1.9%), Br⋯Br (1%), Br⋯C (0.5%) contacts. For reference, the percentage contributions of the *m*'Br*p*CH_3 intermolecular contacts to the total Hirshfeld surface are: H⋯H (36.8%), C⋯H/H⋯C (32.2%), Br⋯H/H⋯Br (15.1%), O⋯H/H⋯O (9.1%), C⋯C (3.4%), O⋯C/C⋯O (1.9%), Br⋯Br (1%), C⋯Br/Br⋯C (0.5%).

SI Table 1: H ••• O Close Contact Distances (Dolomanov *et al.*, 2009). The symmetry codes apply to those molecules interacting with the asymmetric unit. Symmetry Codes m'CNpCH$_3$: (i) *x, 1+y, z*; (ii) *-x, 1-y, 1-z*; (iii) *1-x, 1-y, 1-z.* Symmetry Codes *m*'Br*p*CH$_3$: (i) *1+x, y, z*; (ii) 1-*x, 2-y, 1-z*; (iii) *1-x, 1-y, 1-z*.

***m*'CN*p*CH$_3$ Interactions**	**Distance (Å)**	***m*'Br*p*CH$_3$ Interactions**	**Distance (Å)**
O1 ••• H9^{i}	3.0903 (7)	O1 ••• H9^{i}	2.7778 (13)
O1 ••• H2^{i}	2.8264 (8)	O1 ••• H2^{i}	2.9188 (13)
O1 ••• H15^{i}	2.7457 (7)	O1 ••• H15^{i}	3.1689 (14)
C9–H9 ••• O1^{ii}	3.3415 (7)	C9–H9 ••• O1^{ii}	3.1147 (15)
C15–H15 ••• O1^{iii}	3.1099 (8)	C15–H15 ••• O1^{iii}	3.0001 (14)

List of Symmetry Operators

m'Br*p*CH$_3$

Number	Symm. Op.	Description	Detailed Description	Order	Type
1	x,y,z	Identity	Identity	1	1
2	-x,-y,-z	Inversion center	Inversion at [0, 0, 0]	2	-1

m'CN*p*CH$_3$

Number	Symm. Op.	Description	Detailed Description	Order	Type
**1**	x,y,z	Identity	Identity	1	1
**2**	-x,1/2+y,1/2-z	Screw axis (2-fold)	2-fold screw axis with direction [0, 1, 0] at 0, y, 1/4 with screw component [0, 1/2, 0]	2	2
**3**	-x,-y,-z	Inversion center	Inversion at [0, 0, 0]	2	-1
**4**	x,1/2-y,1/2+z	Glide plane	Glide plane perpendicular to [0, 1, 0] with glide component [0, 0, 1/2]	2	-2

IUPAC Names

4-cyano-2'-fluorochalcone (LERXOW): 3-(4-Cyanophenyl)-1-(2-fluorophenyl)prop-2-en-1-one
4-cyano-4'-diethylaminochalcone (NAWCEU): 4-((1E)-3-(4-(diethylamino)phenyl)-3-oxoprop-1-enyl)benzonitrile
3'-cyano-4-methylchalcone: 3-[(2*E*)-3-(4-methylphenyl)prop-2-enoyl] benzonitrile

4'-bromo-4-methylchalcone (IZEPOI): 1-(4-Bromophenyl)-3-(4-methylphenyl)prop-2-en-1-one
3'-bromochalcone (CICLUW): (2E)-1-(3-Bromophenyl)-3-phenylprop-2-en-1-one
3-bromo-4'-methylchalcone (IGAPAI): (2E)-3-(3-Bromophenyl)-1-(4-methylphenyl)prop-2-en-1-one
3'-bromo-4-methylchalcone: (2*E*)-1-(3-bromophenyl)-3-(4-methylphenyl)prop-2-en-1-one

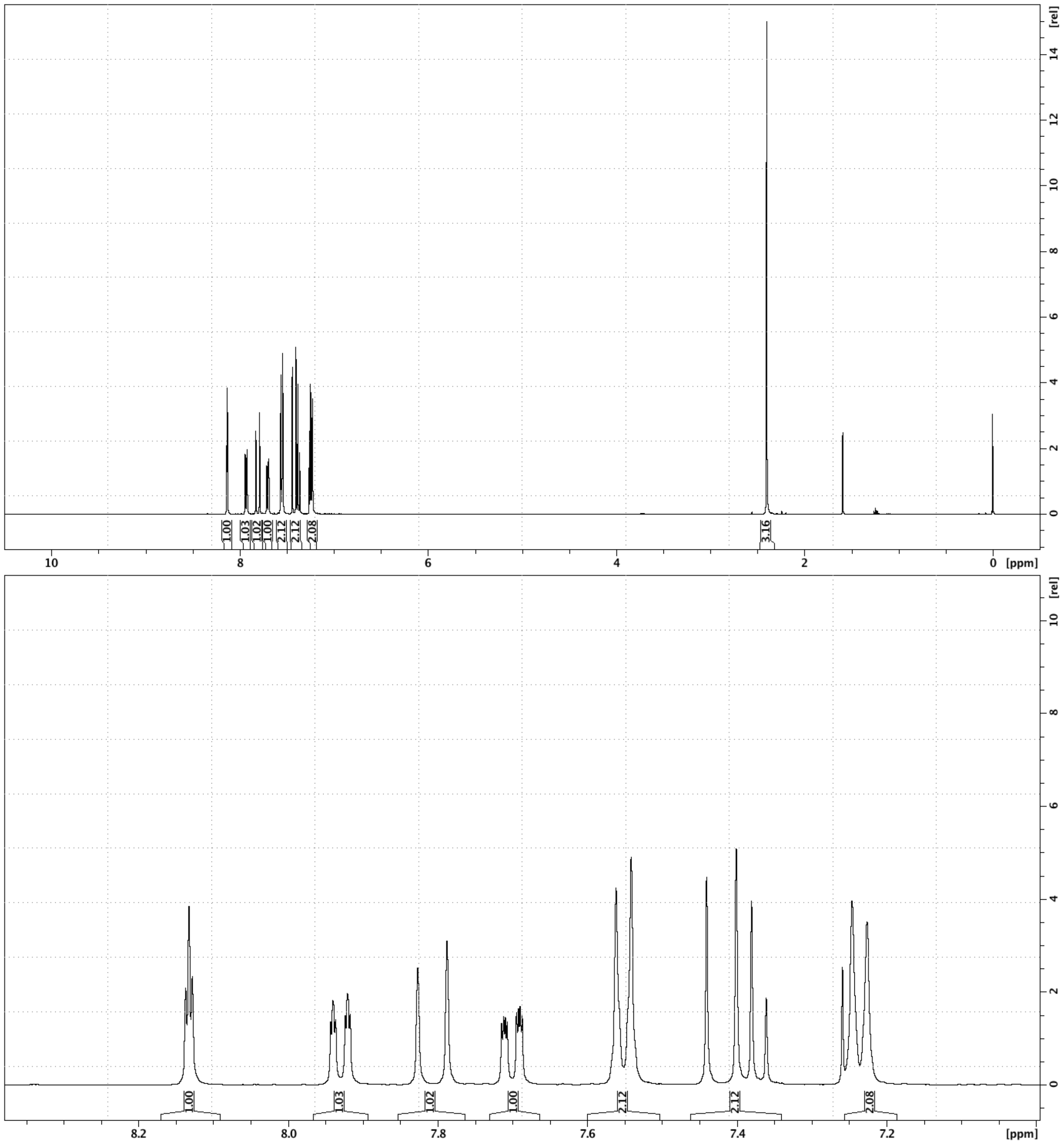

SI Figure 3. ^{1}H NMR spectrum of the material used for crystallization of *m*’Br*p*CH_3. Note the residual $CHCl_3$ (7.26 ppm), H_2O (1.6 ppm), and TMS (0.0 ppm). Integrated signals are for *m*’Br*p*CH_3.

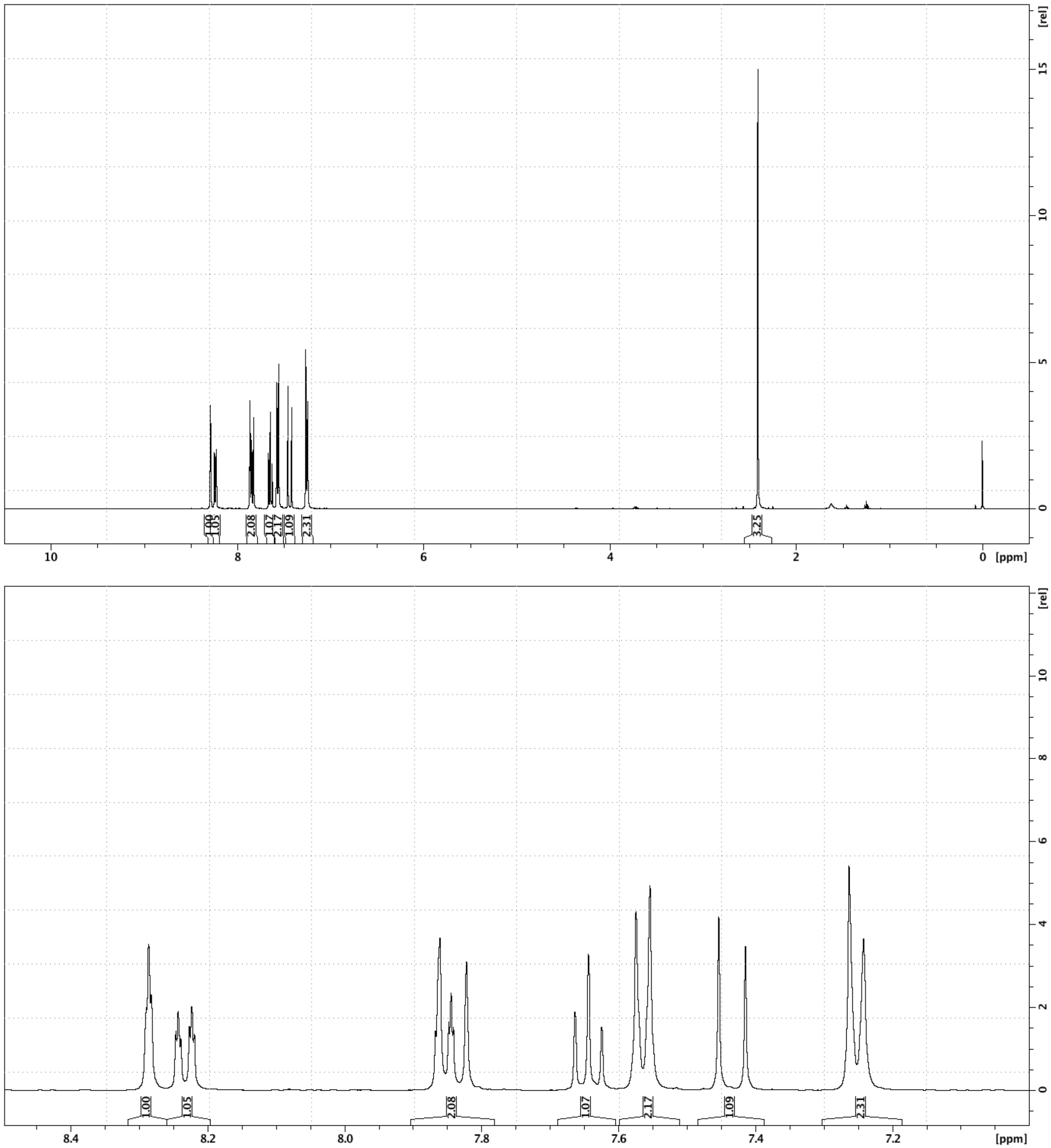

SI Figure 4. ^{1}H NMR spectrum of the material used for crystallization of *m*'CN*p*CH_3. Note the residual $CHCl_3$ (7.26 ppm), H_2O (1.6 ppm), and TMS (0.0 ppm). Integrated signals are for *m*'CN*p*CH_3.
